# Household food insecurity is associated with child’s dietary diversity score among primary school children in two districts in Ghana

**DOI:** 10.29219/fnr.v66.7715

**Published:** 2022-01-07

**Authors:** Janet Antwi, Esi Quaidoo, Agartha Ohemeng, Boateng Bannerman

**Affiliations:** 1 Department of Agriculture, Nutrition and Human Ecology, Prairie View A&M University, Prairie View, TX, USA; 2 Department of Nutrition, School of Public Health and Health Sciences, University of Massachusetts Amherst, Amherst, MA, USA; 3 Department of Nutrition and Food Science, University of Ghana, Legon, Accra, Ghana; 4 Nutrition Linkages Project, University of Ghana, Accra, Ghana

**Keywords:** food insecurity, nutrition knowledge, dietary diversity, school-age children

## Abstract

**Background:**

Dietary diversity is generally considered as a good indicator of nutrient adequacy and is influenced by various factors at the national, household, and individual levels.

**Objective:**

The present study sought to determine the relationships between household food insecurity, primary caregivers’ nutrition knowledge, and dietary diversity of school-aged children in Ghana.

**Methods:**

This forms part of a longitudinal study conducted in the Ayawaso West Municipal district in Accra (urban setting) and the Upper Manya Krobo district (rural setting) in Ghana. Data were collected from a total of 116 caregiver-child dyads using 24-h dietary recall and a short version of the US 12-month Household Food Security Survey Module. Nutrition knowledge and sociodemographic data were obtained using a structured questionnaire. Multivariable logistic regression was used to check for factors associated with children’s dietary diversity.

**Results:**

Majority of households reported food insecurity, with a higher percentage of insecure households located in the rural area (88.9% vs. 46.5%, *P* ≤ 0.0001), compared to the urban setting. Diet diversity among the study children was low, with a mean (standard deviation [SD]) of 5.8 (2.1) out of 14 food groups. Children living in food insecure households were three times more likely to have received low diverse diet compared to those from food secure households (adjusted odds ratio [OR] =3.3, 95% confidence interval [CI]: 1.4–8.0). Caregivers’ nutrition knowledge was, however, not related to children’s dietary diversity.

**Discussion and conclusion:**

Household food insecurity was a main predictor of dietary diversity among school-age children in this study. Thus, caregiver knowledge in nutrition may not be enough, particularly in the presence of food insecurity to guarantee adequate nutrition for school-aged children.

## Popular scientific summary

- Among school going children aged 6-12 years in selected urban and rural settings in Ghana, dietary diversity was generally low
- Household food insecurity was found to be negatively associated with the children’s dietary diversity.
- In line with working to meet Sustainable Development Goal 2, more measures are needed to tackle malnutrition of school age children, paying more attention to factors that influence household level food availability, accessibility, and quality.

Studies on child nutrition have applied several assessment tools to gauge the presence and extent of malnutrition within different communities (1–6). One such tool used to date is the dietary diversity score (7, 8). Dietary diversity can be used as a suitable indicator of nutrient adequacy (7, 9), which reflects one’s nutritional status. Research shows that a child who consistently consumes a variety of meals with basic macro- and micro-nutrients is more likely to meet recommended nutrient intakes that help maintain good health (10). Factors that can influence household and individual dietary diversity are varied and include nutrition knowledge (11, 12), socioeconomic factors (13–16), cultural factors (3, 17), and food security (3, 13, 18). Knowledge and an understanding of nutrition can play a role in caregivers’ food ingredient sourcing, portion-sizing of the various food groups, and meal preparation techniques (11, 12, 19). Socioeconomic factors such as occupation and level of education have also been identified as determinants of dietary diversity, with lower levels of education correlated with low dietary diversity (2).

Food security has four main dimensions: food availability, economic and physical access to food, food utilization, and stability (20), and thus, it is a concept that is closely linked with a child’s ability to consume a diverse diet. Causes of food insecurity in low-income countries include poverty, ineffective food policies, inauspicious climate events, insufficient food production, and difficulty in accessing food due to poor transportation infrastructure (14, 21, 22).

School-age children (typically 6-12 year olds), and younger children, have a high risk of becoming malnourished when their diets are not optimal (23, 24). Research in child nutrition has largely focused on children below the age of 5 years in Ghana. The few published studies that have documented school-age children nutrition situation suggest that this is another vulnerable group that needs attention if the country is to overcome hunger and all forms of malnutrition (25, 26). Information gathered from such research is needed to assist in constructing contextual food policies aimed at promoting adequate nutrition for school-age children at various administrative levels. Therefore, the objective of the parent study from which data were obtained for this current study was to evaluate the effect of a nutrition education intervention on nutrition knowledge, diet, and nutritional status of school-aged children in urban and rural settings in Ghana (26). The study also engaged the children’s caregivers in assessing nutritional knowledge, attitudes, and practices. This current study focuses on the factors that are associated the dietary diversity scores of school-aged children.

## Methods

### Study design

The analysis presented in this paper focused specifically on primary school children and their caregivers for whom data were available in the intervention arm of a parent study conducted to determine the impact of a 6-week nutrition education intervention on the nutrition knowledge, attitudes, and practices of school-age children in Ghana (26). The participants in the intervention arm were included because only caregivers of children in this arm were included in the parent study, and thus, no food insecurity data were available for children in the control group. The baseline data from the intervention study were used for this paper, to exclude any potential effect of the intervention on the variables of interest.

### Study area

This study was conducted in Dzorwulu (urban setting), and Brepaw Upper and Fefe (rural setting) from June through December 2018. Dzorwulu is a vicinity with well-planned residential areas that accommodates the working class and upper crust of Accra society in the Ayawaso West Municipal District, of the Greater Accra Region of Ghana. On the other hand, Brepaw Upper and Fefe are two villages in the Aseseswa subdistrict of the Upper Manya Krobo district in the Eastern Region of Ghana.

### Study population and sampling

For this paper on baseline data of the intervention arm, school children from two conveniently selected public basic schools (one urban and one rural) and their primary caregivers were included. The details of sampling and recruitment processes for the parent study have been described in a previous publication (26). Each arm of the parent study consisted of one urban school and one rural school. The sample size for the parent study was calculated using a 95% confidence rate, 4% error margin, and 80% power to detect a 10% difference (*P* < 0.05) in proportion in nutrition knowledge between intervention and control groups. This gives a minimum required sample size of 86 in each study arm. This was adjusted to 100 per group to account for nonresponses. Caregivers of the children were informed about the study at a Parent Teacher Association meeting, in addition to letters and informed consent forms that were sent home with eligible children for approval. Children within the target age group (6–12 years) who returned signed informed consent forms from their caregivers were included in this study. Caregivers with children within the target age group (6–12 years) who signed informed consent forms for themselves and their children’s participation and gave them to the children to return to the research team were, thus, included in this study.

### Data collection

All study questionnaires were pretested among individuals with characteristics similar to the study population to ensure that the assessments were contextual. Thus, questionnaire pretesting took place in two locations: one school located in an urban area and a second school in the rural setting. Research assistants received a 3-day training in implementing and administering the study tools. Sociodemographic information that was obtained included child’s age, gender, and current class in school. Caregivers were invited to their wards’ schools to interact with the research team. Research assistants interviewed caregivers individually; the interview took approximately 30 min. Data on caregivers’ occupation, marital status, level of education, residence, nutrition knowledge, and household food security were also collected. Information on the dietary intake of the school children was also collected.

Caregivers’ nutrition knowledge was obtained through interview questions that focused on number of meals to feed child, what constituted healthy eating, nutrient content of foods, food safety, quality of food to prevent illness, and food function. To generate knowledge scores for each caregiver, a correct answer to each question was assigned a value of one, and an incorrect answer was coded as zero. Next, all scores under this section were summed up, and the total value was used to represent the knowledge score for that person. The maximum possible score for the nutrition knowledge assessment was 20.

The dietary intake data were gathered using single 24-h dietary recall. Each child was asked to list and describe all foods and beverages consumed at school and at home in the past 24-h indicating the time and source of the food. Visual household measures and food models were used to help children estimate the amounts of foods and beverages consumed (data on food quantities are not presented in this paper). The 24-h recall information was used to calculate dietary diversity scores using the Food and Agriculture Organization guidelines (7), by categorizing food items consumed by the children into 14 food groups. For each food group, a child was given a score of one if he/she consumed any food item in that group, and a score of zero if child did not consume any item in the group. A sum of the scores for all the food groups represented the dietary diversity score of participants. To categorize the dietary diversity variable, the median score of the children computed to be six was used as the cut-off point. Thus, low diet diversity in this study was defined as having a total score less than six, and high dietary diversity was defined as a total score equal or more than six.

The household food security status was measured using the 6-item short form of the US 12-month Household Food Security Survey Module (27). The shorter version was used in order to reduce participants’ response burden, in lieu of the longer version. Despite its length, it has been demonstrated to measure and differentiate food security and food insecurity with sufficient sensitivity, specificity, and minimum bias compared to the lengthy module (28). The affirmative responses (‘often true’, ‘sometimes true’, ‘almost every month’, ‘some months but not every month’, and ‘yes’) were coded as ‘1’ and negative responses as ‘0’, and these were added to get a total score for each household. Scores of 0–1 were classified as food secure, while 2–4 and 5–6 were classified as food insecurity without hunger and food insecurity with hunger, respectively. Households were further classified as food secure (score ≤ 1) and food insecure (score > 1).

### Statistical analysis

All sociodemographic characteristics of the study participants underwent descriptive analyses. The Pearson’s chi-square test for proportions was first used to evaluate the possible relationship between factors including household food insecurity and dietary diversity, as well as socioeconomic factors including child’s age, sex, and caregiver education. The dependent variable of interest was whether a child received a high or a low diversified diet in the 24 h prior to the interview, and the main independent variable of interest was household food insecurity status. Multivariable logistic regression modeling was conducted after the Pearson chi-square test to assess the relationship between household food insecurity status and child’s dietary diversity, adjusting for caregiver nutrition knowledge, formal education, and sociodemographic factors such as the age and sex of children. The choice of variables that were included in the adjusted analysis was based on existing literature on the possible related factors of individual dietary diversity. Although the ‘location’ variable (urban/rural) was significantly associated with dietary diversity (*P* < 0.0001) in the bivariate analysis, it was not included in the adjusted model because of the very low level of variability observed in the sample (only two children in the rural setting had high diverse diet). Statistical analyses were performed using SPSS version 20.0, and *P*-value of <0.05 was considered statistically significant.

### Ethical approval

This study was conducted according to the guidelines laid out in the Declaration of Helsinki, and researcher received ethical approvals and permissions from all the relevant institutions before data collection started.

## Results

A total of 116 caregiver-child dyads were included in this analysis. The mean age of the children was 9.6 (1.8) years, while that of the caregivers was 38.5 (10.8) years (Table 1). Majority of the caregivers (73.3%) who participated in the study were women, and most participants were the biological mothers and fathers (82.8%) of the study children. Most of the primary caregivers had low level of formal education. Only about one-third of them (31.1%) had gone through the Senior High School level or above. Trading was the most common primary occupation among the caregivers (Table 1), followed by farming and vocational jobs such as dressmaking and hairdressing. It is, however, important to note that with the exception of one participant, all the caregivers who indicated farming as their primary occupation were located in the rural setting.

**Table 1:** Sociodemographic characteristics of study participants (*N* = 116)

Characteristic	Mean (SD)	*n* (%)
Children		
Age (years):	9.61 (1.84)	
6–9		48 (41.4)
10–12		68 (58.6)
Sex:		
Male		53 (45.7)
Female		63 (54.3)
School level^a^:		
Lower primary		60 (51.7)
Upper primary		56 (48.3)
Takes money to school:		104 (89.7)
Amount (GH¢):	2.37 (1.85)	
Caregivers		
Age (years)	38.89 (10.79)	
Sex:		
Male		31 (26.7)
Female		85 (73.3)
Marital status:		
Married/co-habiting		86 (74.2)
Separated/divorced/widowed		15 (12.9)
Single		15 (12.9)
Formal education^b^:		
None		23 (19.8)
Primary		16 (13.8)
JHS		41 (35.4)
SHS		27 (23.3)
Above SHS		9 (7.7)
Primary occupation:		
Trading		40 (34.5)
Farming		26 (22.4)
Vocational		26 (22.4)
Unemployed/student		5 (4.3)
Other^c^		19 (16.4)
Relation to study child:		
Parent		96 (82.8)
Other relative		20 (17.2)
Estimated household income/month (GH¢):		
≤500		71 (61.2)
Above 500		35 (30.2)
Do not know		10 (8.6)
Residence:		
Urban		71 (61.2)
Rural		45 (38.8)

a Lower primary consists of classes One to Three, while Upper primary is made up of classes Four to Six.

b JHS represents Junior High School, and SHS represents Senior High School in Ghana.

c Other occupations included public servants, pensioners, domestic help, and laborer. Values are presented as frequencies (%) or means (standard deviation).

**Fig. 1:**
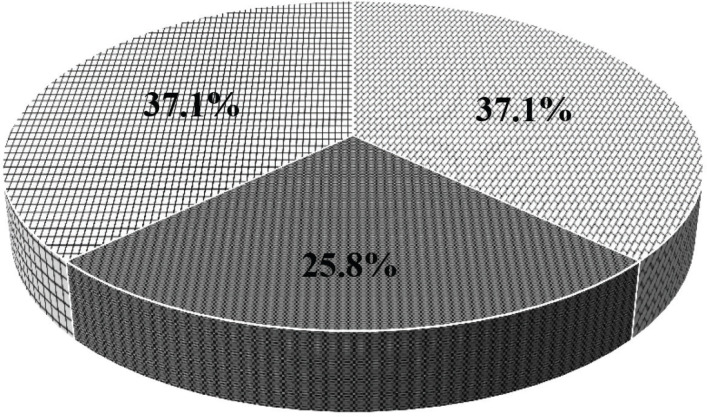
Household food insecurity among study participants. The darkest section represents study respondents who were food insecure without hunger. The crossed-line section represents study respondents who were food insecure with hunger, and the brick section represents the study respondents who were food secure.

The experience of household food insecurity was reported by majority of the caregivers (62.9%), with only about one-third being classified as food secure (Fig. 1). Of households that were classified as food insecure, more than half were severely food insecure. Food insecurity was reported by a higher proportion of caregivers in the rural setting (88.9% vs. 46.5%, *P* ≤ 0.0001), compared to those in the urban setting.

**Fig. 2:**
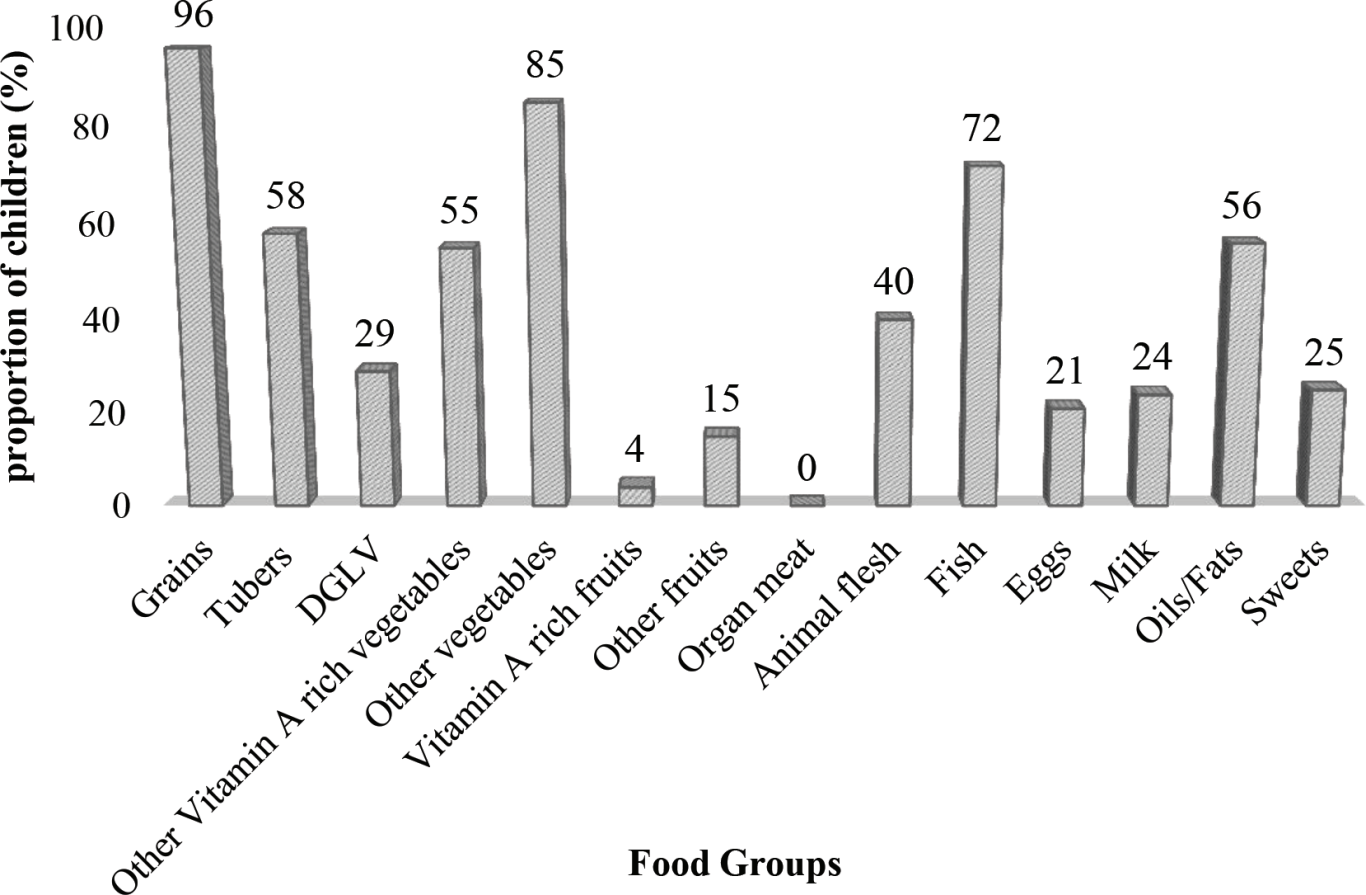
Intake of food groups by the school children, based on a single 24-h dietary recall. Bars represent the groups of food that the school children ate in 24 h. DGLV stands for dark green leafy vegetables.

Based on a single 24-h recall, the most consumed food items consumed by the children were from the grains’ food group, followed by the ‘other vegetable’ group such as onions and garden eggs (Fig. 2). Among the animal source foods, the most common type consumed by the study children during the period of assessment was fish, but organ meat was totally absent from their diet. Notably, vitamin A rich fruits were the least consumed in the vegetable and fruit category.

Generally, diet diversity among the study children was low, with a mean score of 5.8 (2.1) out of 14 food groups. Using the group median of six as cut-off, about half of the study children (50.9%) consumed high diverse diets within the 24 h prior to the data collection. Bivariate analysis indicated a higher proportion of older children received diverse diet compared to younger children, but there was no difference based on gender (Table 2). Almost all children (96.2%) living in households, where the main occupation of the primary caregiver was farming, received low diverse diet within the period of observation. In a logistic regression model, dietary diversity of the children was significantly associated with household food insecurity and child’s age (Table 3). Children living in food insecure households were three times more likely to have received a low diverse diet, and this was significant for both levels of food insecurity (without and with hunger), compared to children from food secure households. On the other hand, older children were less likely to have eaten a low diverse diet (odds ratio [OR] = 0.8, 95% confidence interval [CI]: 0.6 – 0.9). There was also a tendency for children whose primary caregivers had formal education up to at least Senior High to consume a high diverse diet. There was, however, no significant association between dietary diversity of children and caregivers’ nutrition knowledge.

**Table 2:** Bivariate analysis comparing study school children based on dietary diversity

Independent variables	Child’s dietary diversity			*P*
	Total	High (*n* = 59)	Low (*n* = 57)	
Child’s sex				0.270
Male	53 (45.7)	24 (45.3)	29 (54.7)	
Female	63 (54.3)	35 (55.6)	28 (44.4)	
Child’s age				0.041
6–9 years	48 (41.4)	19 (39.6)	29 (60.4)	
10–12 years	68 (58.6)	40 (58.8)	28 (41.2)	
Caregiver education				0.022
Below SHS	80 (69.0)	35 (43.8)	45 (56.2)	
SHS and above	36 (31.0)	24 (66.7)	12 (33.3)	
Caregiver occupation				0.002
Trader	40 (34.5)	23 (57.5)	17 (42.5)	
Farmer	26 (22.4)	1 (3.8)	25 (96.2)	
Vocational	26 (22.4)	17 (65.4)	9 (34.6)	
Unemployed	5 (4.3)	4 (80.0)	1 (20.0)	
Other	19 (16.4)	14 (73.7)	5 (26.3)	
Area of residence				<0.0001
Urban	71 (61.2)	57 (80.3)	14 (19.7)	
Rural	27 (38.8)	2 (4.4)	25 (95.6)	
Household food security				0.006
Food secure	43 (37.1)	29 (67.4)	14 (32.6)	
Food insecure	73 (62.9)	30 (41.1)	43 (58.9)	

Data presented as frequency (%).

**Table 3:** Factors associated with child diversity as unadjusted and adjusted odds ratios

Independent variables	Low child dietary diversity			
	Unadjusted		Adjusted	
	OR	95% CI	OR	95% CI
Child’s age (years)	0.8	0.7, 1.0	0.8	0.6, 0.9
Child’s sex	0.7	0.3, 1.4	0.7	0.3, 1.6
Male, female^a^				
Caregiver education	0.4	0.2, 0.9	0.4	0.2, 1.1
Below Senior High (SHS) Level^a^				
SHS and above				
Caregiver nutrition knowledge	1.1	0.8, 1.4	1.2	0.9, 1.7
Household food security				
Food secure^a^	3.1	1.2, 8.2	2.9	1.0, 8.4
Food insecure without hunger	2.9	1.2, 6.9	3.1	1.2, 8.0
Food insecure with hunger				

Using the group median of 6 as cut-off, low diversity was defined as a total diversity score less than 6. Diversity score was calculated according to FAO guidelines (7).

a Reference category of the categorical variables ^§^*P* < 0.10 and *P < 0.05.

## Discussion

Engaging caregivers in an attempt to piece together school children’s nutrition situation is particularly important since key determinants of dietary practices adopted by children include both caregivers’ and household characteristics. Our study engaged caregivers of school aged children in urban and rural Ghana to assess their nutrition knowledge and their households’ food security.

Dietary diversity, in general, was low among both urban- and rural-based school aged children. Consumption of grains was high among our sample of children with rural-based children consuming more grains than any other food group when compared to their urban-based counterparts. This finding is consistent with that from a study in Uganda (29), indicating that grain consumption is generally high among children in sub-Saharan Africa as they form most of the staple foods. Starchy tubers and roots were also largely consumed by both urban- and rural-based children. Starchy foods are an indispensable meal ingredient in many African meals as they seem to be readily available and accessible than other food staff (8, 30). Grains and starches are relatively cheaper than other food groups such as animal protein, and starch-based meals are perceived to be quenchers of hunger (15, 30). On the other hand, low consumption of fruits, vegetables, animal protein, and dairy was observed among participants, similar to other studies (29–31). One may expect that with majority of rural households in this study engaged in farming, intake of vegetables in general would be high due to the cultivation of these food items on their farms. However, it has been noted that subsistence farmers in developing countries focus on growing few varieties of crops, which are mainly slated for sale (29, 30). As a result, farming households tend to base their diets on few food groups resulting in low dietary diversity of household members. Additionally, reports indicate that in developing countries, many crops are consumed only when they are in season, particularly in households with low incomes (32, 33). In this study, dark green leafy vegetables (DGLV), a rich source of iron, folate, and beta-carotenes, were not consumed by most of the children. Even though our study did not collect data on seasonal variations in relation to dietary intake, this research took place when common local DGLV was out of season. Considering that most of the households had low incomes, this seasonality might have accounted for the low intake of nutrient-dense dark leafy vegetables among the school children in this study.

Our study identified more than half of the study’s participating households as food insecure, with about a quarter of them identified as being food insecure with hunger, and household food insecurity was strongly associated with child dietary diversity. Earlier studies have reported varied prevalence of food insecurity in different parts of Ghana (18, 34, 35), indicating vast differences with respect to different parts of the country as well as the seasonality of food availability. Additionally, almost all farming households were food insecure in the current study. This is similar to findings from study that assessed household food security in farming and nonfarming communities in three ecological regions in Ghana (18). Many of the rural-based caregivers did not have a steady income, relying largely on the sale of produce from their farms to gain income. In the urban setting, most of the caregivers reported earning monthly incomes of less than 500 Ghana cedis (i.e. 85.8 US dollars). Household wage earners with regular incomes have more purchasing power and may be more likely to purchase food items even when they are out of season and not as common (2) and, thus, can vary the meals consumed at the household level, compared to households where income is not as steady (20). It is also important to note that the observed association between household food insecurity and child dietary diversity in this study has also been noted among younger children (18, 20). Thus, there is the need to include food security issues at the household level as an integral part of policies and strategies addressing nutrition of all children.

There was a tendency for children whose primary caregivers had less than secondary education to receive low diverse meals during the observation period. A study in Algeria reported that the educational level of caregivers played a significant role in the dietary diversity of children in their study (36). Considering that education qualification can influence caregivers’ income (2), the higher a caregiver’s education level, the more likely a household would have high household income, which could translate into a more diversified diet for children within the household.

Caregivers’ nutrition knowledge, however, was not associated with dietary diversity of the school children studied, contrary to other studies (11, 12). It has been suggested that mothers who have knowledge on nutrition have a protective impact on their children’s nutrition status as they are better equipped to make appropriate dietary decisions for their wards (19). However, caregiver knowledge alone is insufficient to result in better diet since other important factors such as food availability and accessibility and intra-household food allocation influence what is finally consumed. For example, milk and milk products were barely consumed by the children in the current study, even though some caregivers knew the benefits of dairy. Thus, for our study sample, caregivers’ knowledge of nutrition did not necessarily translate into diverse diet. This study illustrates that even though an awareness of the advantages of specific foods to children’s health may be present in a caregiver, the availability, accessibility, and utilization of these products would be an issue in a food insecure home. Thus, food insecurity, as observed in this study, can have a greater impact on the dietary choices made for children than parental awareness of adequate nutrition behaviors.

## Conclusion

The findings from this study indicate a significant relationship between household food security and dietary diversity of school age children, but caregivers’ nutrition knowledge was not associated with diet diversity of the children. Our study provides additional evidence that various factors such as household level food security play important roles in ensuring good quality nutrition for school-aged children. Programmes aimed at tackling malnutrition among school-aged children should, therefore, tailor interventions that address numerous drivers of child malnutrition including food insecurity at household levels.

### Study limitations

These findings should be interpreted with some level of caution. This study establishes possible associations, not causality. Although the researchers used validated questionnaires and rigorous assessment tools, not all factors that could possibly impact a child’s dietary diversity, such as cultural practices and seasonal variations of food availability, were assessed. Additional information on the actual nutrient quality of the meals consumed by the children was not recorded.
